# Correlates of Marijuana Drugged Driving and Openness to Driving While High: Evidence from Colorado and Washington

**DOI:** 10.1371/journal.pone.0146853

**Published:** 2016-01-22

**Authors:** Kevin C. Davis, Jane Allen, Jennifer Duke, James Nonnemaker, Brian Bradfield, Matthew C. Farrelly, Paul Shafer, Scott Novak

**Affiliations:** RTI International, Research Triangle Park, NC, United States of America; Mario Negri Institute for Pharmacological Research and Azienda Ospedaliera Ospedali Riuniti di Bergamo, ITALY

## Abstract

**Aims:**

A potential unintended consequence of legalizing recreational marijuana is increased marijuana-related driving impairment. Some states where recreational marijuana is legal have begun implementing interventions to mitigate driving under the influence (DUI) of marijuana, including media campaigns to increase knowledge about DUI laws. However, little is known about the associations between knowledge of DUI laws and marijuana DUI behavior. In this study, we provide new data from a survey of marijuana users in Colorado and Washington to examine associations between marijuana drugged driving and two potential behavioral precursors of marijuana DUI. We also explore other factors that may influence marijuana DUI.

**Methods:**

Data are from an online survey of marijuana users in Colorado and Washington. Respondents who reported any marijuana use in the past 30 days (n = 865) served as the analytic sample. We examined prevalence of two behavioral outcomes: (1) any driving of a motor vehicle while high in the past year and (2) driving a motor vehicle within 1 hour of using marijuana 5 or more times in the past month. Additional outcomes measuring willingness to drive while high were also assessed. Logistic regressions were used to estimate each outcome as a function of two multi-item scales measuring knowledge of the legal consequences of driving high and perceptions that driving while high is not safe. Additional covariates for potential confounders were included in each model.

**Results:**

Prevalence of past-year driving while under the influence of marijuana was 43.6% among respondents. The prevalence of driving within 1 hour of using marijuana at least 5 times in the past month was 23.9%. Increased perception that driving high is unsafe was associated with lower odds of past-year marijuana DUI (OR = 0.31, *P* < 0.01) and lower past-month odds of driving 5 or more times within 1 hour of using marijuana (OR = 0.26, *P* < 0.01). Increased knowledge of marijuana DUI laws was also associated with lower odds of each of these outcomes (OR = 0.63, *P* < 0.01, OR = 0.69, *P* = 0.02, respectively). Post-estimation Wald tests confirmed the negative associations with marijuana DUI were greater in magnitude for safety perceptions than knowledge of DUI laws. Increased perceptions that driving while high is unsafe was associated with significantly lower willingness to drive after using marijuana while increased knowledge of marijuana DUI laws was not associated with these outcomes.

**Conclusions:**

Despite recent interventions targeting public awareness of the legal consequences of marijuana DUI, our results suggest that knowledge of these laws is a weaker predictor of DUI behavior than perceptions that driving high is unsafe. In addition, safety perceptions predict decreased openness to driving high while knowledge of DUI laws was not associated with openness. These findings suggest that interventions for reducing the incidence of marijuana DUI are likely to be more successful by targeting safety perceptions related to marijuana DUI rather than knowledge of DUI laws. We caution that because these data are limited to an online convenience sample, results may not be generalizable beyond our sample.

## Introduction

In 2012, Colorado and Washington became the first states to legalize retail sales of recreational marijuana to adults ages 21 and older. Sales at retail dispensaries began on January 1, 2014, in Colorado and on July 8, 2014, in Washington. In the November 2014 elections, Oregon and Alaska voted to legalize the sale of recreational marijuana, with each state expecting to start accepting retail licenses in late 2015 and early 2016, respectively. The District of Columbia also recently voted to legalize home production and possession of small amounts of recreational marijuana. Numerous other states are considering ballot initiatives in the near future to legalize recreational marijuana [1]. These trends in adoption of legal recreational marijuana and previous adoption of legal medical marijuana have raised concerns about unintended consequences on public health and safety.

A primary potential unintended consequence of legalizing recreational marijuana is increased marijuana-related driving impairment. In Colorado and Washington, penalties for being caught driving while high include being charged with driving under the influence (DUI), loss of driver’s license, and fines, among others. Among drivers in the U.S. National Roadside Survey, positive tests for delta-tetrahydrocannabinal (THC) increased from 8.6% in 2007 to 12.6% in 2013 and 2014, representing a relative increase of 47%. In this study, marijuana showed the largest increase of all drugs tested [2]. Previous research also strongly suggests that marijuana use impairs the ability to drive [3, 4], particularly among occasional marijuana users who may be less tolerant to THC [5]. A recent review of evidence on marijuana-related driving impairment by Hartman and Huestis [5] summarizes a range of studies with experimental designs that have demonstrated the effects of marijuana on drivers’ road tracking, lane position variability (i.e., swerving), and steering wheel variability, among other metrics.

Despite the aforementioned studies showing links between marijuana use and impaired driving, the relationship between marijuana use and actual motor vehicle accidents is less clear. A recent widely publicized study by the National Highway Traffic Safety Administration (NHTSA) [6] used a case-control design to show that, after controlling for age, gender, race/ethnicity, and alcohol use, there were no statistically significant associations between testing positive for THC in blood and being involved in a motor vehicle accident. However, this study was limited to a local sample from Virginia, which did not have legal recreational marijuana or a medical marijuana program at the time of data collection. Other recent studies that focus on states where marijuana has been commercialized (i.e., Colorado and Washington) suggest that the introduction of commercialized marijuana has led to increases in marijuana-related driving impairment. For example, Salomonsen-Sautel and colleagues [7] found that the proportion of marijuana-positive drivers in fatal motor vehicle crashes in Colorado increased significantly after commercialization of medical marijuana. In Washington, Couper and Peterson [8] demonstrate significant increases in the prevalence of marijuana in suspected impaired driving cases since legalization, while the prevalence of alcohol and other drugs in the same population of suspected impaired drivers did not change during the pre-post legalization period.

Although evidence on the relationship between marijuana use and motor vehicle accidents is mixed, there are virtually no data on the role that new marijuana products such as edibles may play in driver impairment. Recent news reports [9, 10] suggest growing concern over the availability of edible marijuana in the new retail environments in Colorado where unanticipated increases in severe intoxication and even deaths related to ingestion of edibles have been documented. A recent viewpoint article published in the *Journal of the American Medical Association* [11] reported unexpected increases in marijuana intoxication among 2,000 weekly patients seen in the emergency room of the University of Colorado’s hospital in Aurora, Colorado. These anecdotal reports further suggest the potential for increases in marijuana-related driver impairment in states where edibles and other potent variants of commercial marijuana are available.

Amid the complexities of implementing retail marijuana, policy makers in states with legal retail marijuana have begun implementing interventions to mitigate unintended consequences related to marijuana use and driving. These interventions have included mass media campaigns aimed at increasing the public’s knowledge about the legal consequences of driving while high as a way to curb marijuana-related DUI. For example, in Colorado, a statewide media campaign called “Drive High, Get a DUI” was aired during the spring of 2014 to remind Coloradans that, while using recreational marijuana was now legal, driving while under the influence of marijuana remained illegal. This campaign was also aired in Washington in the summer of 2014, during the launch of their retail marijuana market.

Given that behavioral intent, perceptions, and other cognitive factors are often predictive of actual behavior change [12, 13], it is important to identify and understand the myriad of risk perceptions, knowledge, attitudes, and beliefs that may precede the behavior of marijuana use while driving. These perceptions and cognitive precursors can be important targets of policy, prevention messaging, and other interventions aimed at ultimately reducing drugged driving with marijuana. For example, the “Drive High, Get a DUI” campaign described above targeted knowledge of marijuana DUI laws under a theoretical premise that increasing knowledge of the legal consequences of driving high will lead to reductions or at least prevent increases in marijuana DUI behaviors and intentions. Perceptions and beliefs about the safety of driving while high may also be important potential targets for interventions of this type. However, state-specific data on the association between these potential behavioral precursors and driving while high are limited. In addition, there is little if any data on demographic and other individual-level correlates of driving while high to inform how such interventions may be effectively targeted to specific populations.

In this study, we provide new data from an online survey of marijuana users in Colorado and Washington that describes the associations between marijuana DUI behavior and two potential behavioral precursors of marijuana DUI: 1) knowledge of the potential legal consequences of driving while under the influence of marijuana; and 2) perceptions of the safety of driving while under the influence of marijuana. We examined the former as a practical test of an actual strategy employed by a previous real-world intervention (“Drive High, Get a DUI” campaign) while the latter is analyzed as a test of broader theoretical predictions about the relationships between attitudes, beliefs, and perceptions and downstream behavioral outcomes [12, 13]. We also compare the magnitudes of these associations to estimate whether one type of behavioral precursor is more predictive of marijuana DUI than the other. In addition, we examine demographic and other individual-level correlates of driving while high to help inform how future interventions may be effectively targeted to specific populations. These data may provide actionable information and insights on the development of future messages and interventions to protect public safety and mitigate increases in marijuana DUI.

## Methods

### Survey data

We surveyed marijuana and hashish users in Colorado and Washington from the Global Market Insite (GMI) panel, an established online panel of U.S. consumers. Respondents were recruited via e-mail invitations that contained a brief description of the study and a link to the online survey. Respondents first completed a brief screening questionnaire that assessed age and recent marijuana use. Those who were aged 18 or older from Colorado or Washington and used marijuana at least once in the past year were eligible to participate. To determine study eligibility, our screening questionnaire assessed ever use, past year use, and past 30-day use of marijuana using item wording from the 2012 National Survey on Drug Use and Health (NSDUH). To yield a sample that more closely resembles current marijuana users, we restricted all analyses in this study to respondents who used marijuana or hashish in the past 30 days.

A total of 7,587 respondents completed the screening questionnaire. Of these, 2,992 respondents qualified for the study as a past-year marijuana user and 1,352 of those consented and completed the full survey. This yielded an overall survey cooperation rate of 45.2% among those who were eligible to participate. Among the 1,352 respondents who completed the survey, 865 were past 30-day users, with 399 from Colorado and 466 from Washington. Past 30-day users serve as the final analytic sample for this study.

Data were collected during the final week of September 2014. All surveys were self-administered online, and the average interview length was approximately 20 minutes. Respondents were compensated with GMI “MarketPoints,” redeemable for online merchandise with a cash value of up to $10. The survey instrument, recruitment procedures, incentives, and all other data collection protocols were reviewed and approved by the sanctioned institutional review board of RTI International.

### Measures

#### Outcome Variables

The primary dependent variables consisted of two dichotomous measures of marijuana drugged driving behavior. The first was an indicator variable for any driving of a motor vehicle while high or feeling the effects of marijuana or hashish in the past year. The second outcome was defined as an indicator variable for driving a motor vehicle within 1 hour of consuming marijuana or hashish 5 or more times in the past month. This construct was chosen to measure more severe and recent marijuana drugged driving and to provide comparability to similar measures of more frequent marijuana DUI behavior used in other recent studies [14]. We also measured two secondary outcomes that capture marijuana users’ willingness to drive while high in the future. These items were measured as dichotomous indicators for answering “definitely yes or probably yes” to each of the items “I might drive high even though I know I shouldn’t” and “In certain situations, I might drive high.” While there are no measures comparable to the latter two outcomes in publicly available surveillance systems, they are based on similar constructs that have been used to measure openness to smoking and other problem behaviors.

#### Independent Variables

Our primary independent variables consisted of two multi-item scales to capture two potential behavioral precursors of marijuana drugged driving: 1) degree of knowledge of the potential legal consequences of driving high; and 2) perceptions the using marijuana while driving is unsafe. Degree of knowledge of marijuana DUI laws was assessed with a 3-item scale where respondents were asked to indicate their agreement with the statements 1) I could get a DUI for driving high; 2) I could lose my license for driving high; and 3) I could pay a fine for driving high. Perception that marijuana drugged driving is unsafe was measured with a 5-item scale consisting of the statements 1) I can safely drive under the influence of marijuana; 2) It is safer driving under the influence of marijuana than under the influence of alcohol; 3) If I am just a little bit high, I don’t think my ability to drive is impaired; 4) Being high on marijuana or hashish doesn’t affect my driving; and 5) Driving high is not a big deal. Each of the scale items were answered on a 5-point Likert-type response scale of (1) strongly agree; (2) agree; (3) neither agree nor disagree; (4) disagree; and (5) strongly disagree. All items in each scale were summed and then divided by the number of items in the scale to return the scale to a continuous metric with the same magnitude as any of the single items. The marijuana DUI law knowledge scale was reverse coded in our analysis so that a value of 1 corresponds to strongly disagree and 5 corresponds to strongly agree. This conversion ensures that positive values of both outcome scales represent the “desired” responses from a societal perspective.

#### Potential Confounders

We measured a range of demographic covariates that may be associated with marijuana drugged driving behaviors and openness. Respondent age was measured with separate indicator variables for ages 25 to 34, ages 35 to 54, and ages 55 and over (ages 18–24 excluded as the reference group). Gender was measured with a simple indicator variable for male (female excluded as reference group) and race/ethnicity was measured with a series of mutually exclusive indicators for non-Hispanic black, Hispanic, and non-Hispanic other races (non-Hispanic white excluded as reference group). Due to small cell sizes for minority races/ethnicities in our data, we include race/ethnicity as a control variable in our analysis but do not make statistical inferences about the associations between race/ethnicity and the outcome variables. We also measured educational attainment with a set of dichotomous indicators for high school graduate, some college, and having at least a 4-year college degree (less than high school graduates excluded as the reference group). In addition, employment status was measured as a dichotomous indicator being either self-employed or employed for wages. Finally, we included an indicator variable for residing in Colorado (Washington excluded as reference) to account for potential differences in marijuana drugged driving and openness to driving while high between each of these two states.

To capture levels of marijuana use, we measured marijuana consumption on the last day it was consumed with the following question: “Now we are going to show you five pictures of marijuana. Thinking about the last day you used marijuana, how much did you personally use that entire day? Please check the box next to the photo that best represents how much you used.” Images showed 1/8 oz., 1/16 oz., 1/32 oz., 1/64 oz., and 1/128 oz. of marijuana. To orient the respondent’s sense of how much each of these amounts represented, each image showed the marijuana next to a 25 cent U.S. quarter. Ounces were used as the unit of marijuana because these are the increments by which they are commonly sold. For purposes of our statistical analysis, we created indicator variables to indicate “moderate” use (1/32 oz. or 1/64 oz.) and “high use” (1/8 oz. or 1/16 oz) with “low use” (1/128 oz. or less) excluded as the reference category in multivariate analysis.

Respondents who answered “don’t know” or who refused to answer specific questions underlying any of the measures described above were treated as missing data and excluded from analysis of those items. The overall rate of missing data on each outcome as well as each covariate we analyzed was less than 3%.

### Statistical analysis

#### Reliability of Item Scales for Primary Independent Variables

We conducted principal factor analysis to assess the construct reliability for each primary independent variable scale measuring knowledge of marijuana DUI laws and perceptions about the safety of driving while high [15]. This was done by using the principal factor method in Stata statistical software to calculate eigenvalues for all identified factors and loadings for retained factors from each scale to verify that each scale yields a single-factor solution. Scale reliability was then assessed by estimating Cronbach’s alpha coefficient for each scale.

#### Multivariate Analysis to Assess Correlates of Marijuana DUI Behaviors and Openness

We used logistic regressions to estimate each dichotomous outcome variable describe previously as a function of the knowledge and safety perception scales. Each scale item was entered into the model simultaneously to facilitate comparisons of the magnitudes of the associations between these predictors and the drugged driving outcomes we measured. These comparisons were formally conducted using post-estimation Wald tests of equality between regression coefficients for each of the primary independent variable scales described previously. In addition, all multivariate models included covariates for the demographic characteristics noted above to assess the associations between these factors and each outcome we measured. All logistic regression coefficients were converted to odds ratios for ease of model interpretation.

## Results

### Sample characteristics

Respondent demographics are summarized in Table 1. Respondents’ age was generally older than the adult population as a whole with 70.2% being aged 35 or older. Females comprised 60.4% of respondents. Respondents were also predominantly non-Hispanic white, making up 79.3% of the combined sample. Approximately 3.4% of respondents in our sample were non-Hispanic black, 7.5% were Hispanic, and 9.8% were non-Hispanic other races/ ethnicities. Educational attainment was relatively high as 29.4% and 55.4% of respondents reported having either some college or a college degree or more, respectively.

**Table 1:** Demographics among Past 30-Day Marijuana Users in Colorado and Washington.

Sample Characteristic	Proportion (n = 865)
Age	
18 to 24	8.7%
25 to 34	21.2%
35 to 54	38.4%
55 or older	31.8%
Gender	
Male	39.7%
Female	60.4%
Race/ethnicity	
White, non-Hispanic	79.3%
Black, non-Hispanic	3.4%
Hispanic	7.5%
Other, non-Hispanic	9.8%
Education	
Did not graduate high school	1.6%
High school or GED	13.6%
Some college, no degree	29.4%
College degree or higher	55.4%

### Reliability of safety perceptions and DUI knowledge scales

Principle factor analysis results for the two primary explanatory variables in our study are summarized in Table 2. Our scale for belief that it is unsafe to drive while using marijuana yielded a single factor solution with factor loadings ranging from 0.70 to 0.87 across the 5 items in this scale. Reliability for of this scale was high with an estimated Cronbach’s alpha of 0.91. The scale was also distributed fairly normally with an overall mean of 3.27 and a standard deviation of 1.15. The 3-item scale we used to measure knowledge of the legal consequences of driving high also yielded a single factor solution with factor loadings ranging from 0.51 to 0.60. Reliability for this scale was more moderate, with an estimated Cronbach’s alpha of 0.63. This scale had a mean value of 4.13 and a standard deviation of 0.82.

**Table 2:** Descriptive Statistics and Reliability Analysis of Scales for Perceptions of Safety of Using Marijuana While Driving and Knowledge of Marijuana DUI Laws.

Belief that it is unsafe to drive high	Mean	Std. Dev	Min	Max	Factor Loading	Alpha
I can safely drive under the influence of marijuana.^a^	3.29	1.40	1	5	0.87	0.87
It is safer driving under the influence of marijuana than under the influence of alcohol.^a^	2.68	1.44	1	5	0.70	0.91
If I am just a little bit high, I don’t think my ability to drive is impaired.^a^	3.24	1.33	1	5	0.85	0.88
Being high on marijuana or hashish doesn’t affect my driving.^a^	3.51	1.29	1	5	0.84	0.88
Driving high is not a big deal.^a^	3.62	1.25	1	5	0.80	0.89
*Test Scale*	*3*.*27*	*1*.*15*	*1*	*5*	*—-*	*0*.*91*
**Knowledge of Legal Consequences of Driving High**						
I could get a DUI for driving high.^b^	4.27	0.96	1	5	0.60	0.49
I could lose my license for driving high.^b^	4.04	1.11	1	5	0.51	0.60
I could pay a fine for driving high.^b^	4.10	1.08	1	5	0.58	0.53
*Test Scale*	*4*.*13*	*0*.*82*	*1*	*5*	*—-*	*0*.*63*

^a^ 1 = strongly agree; 2 = agree; 3 = neither agree nor disagree; 4 = disagree; 5 = strongly disagree.

^b^ 1 = strongly disagree; 2 = disagree; 3 = neither agree nor disagree; 4 = agree; 5 = strongly agree.

### Marijuana DUI behavior and openness

Descriptive statistics for each of our 4 outcome variables are summarized in Table 3. Approximately 43.6% of respondents reported driving a motor vehicle while high or feeling the effect of marijuana or hashish in the past year. The prevalence of driving a motor vehicle within 1 hour of consuming marijuana or hashish at least 5 times in the past 30 days was 23.9% among respondents. Approximately 52.7% of respondents agreed or strongly agreed that they might drive high even though they know they should not. In addition, 41.3% of respondents agreed or strongly agreed that they might drive while high in certain situations.

**Table 3:** Driving while Under the Influence of Marijuana and Openness to Drive While High [95% confidence interval].

Behavior and Openness Outcome	Past 30-daymarijuana users(n = 865)
Percentage of marijuana users who report driving a car or other motor vehicle while high or feeling the effect of marijuana or hashish in past year	43.6% [40.3–47.0]
Percentage of marijuana users who report 5 or more times in the past 30 days driving a motor vehicle within 1 hour of consuming marijuana or hashish	23.9% [20.3–27.6]
Percentage of marijuana users who agree or strongly agree that “I might drive high even though I know I shouldn’t.”	52.7% [49.3–56.1]
Percentage of marijuana users who agree or strongly agree that “In certain situations, I might drive high.”	41.3% [37.9–44.6]

### Association between marijuana safety perceptions and knowledge of DUI laws and marijuana DUI behavior and openness

Logistic regression results from our models for associations between each outcome variable and the explanatory variables of marijuana DUI safety perceptions and knowledge of DUI laws are summarized in Table 4. Results of these models show that increased beliefs that it is unsafe to drive while high is associated with a lower odds of any driving while under the influence of marijuana in the past year (OR = 0.31, *P* < 0.01). Increased knowledge of the legal consequences of driving while under the influence of marijuana was also associated with decreased odds of any driving while under the influence of marijuana in the past year (OR = 0.63, *P* < 0.01). Post-estimation Wald tests confirm that the magnitude of the negative association with any driving while under the influence of marijuana in the past year is significantly larger for the safety perceptions scale than the marijuana DUI knowledge scale (*P* < 0.01). Model results further show that increased perception that it is unsafe to drive while high is also associated with a lower past-month incidence of driving 5 or more times within 1 hour of consuming marijuana or hashish (OR = 0.26, *P* < 0.01). Knowledge of marijuana DUI laws was also associated with past-month incidence of this outcome (OR = 0.69, *P* = 0.02). Post-estimation Wald tests show that the magnitude of the negative association with this outcome is also significantly larger for the safety perceptions scale than for the marijuana DUI knowledge scale (*P* < 0.01).

**Table 4:** Odds Ratios (*P* values) Showing Odds of Marijuana DUI Behaviors and Openness as a Function of Safety Perceptions, Knowledge of DUI Laws, and Respondent Characteristics,

	Marijuana DUI Behaviors		Openness to Driving While High	
Independent Variable	Drove car or other vehicle while high in past year	Drove while high 5 or more times in past month	In certain situations, I might drive high^1^	I might drive high even though I know I shouldn’t^1^
Perception that driving while high is unsafe (scale)	0.31 (< 0.01)	0.26 (< 0.01)	0.21 (< 0.01)	0.25 (< 0.01)
Knowledge of marijuana DUI laws (scale)	0.63 (< 0.01)	0.69 (0.02)	1.23 (0.10)	1.02 (0.88)
Marijuana consumption on last day used (Reference: 1/128 oz or less)				
1/64 oz to 1/32 oz	1.51 (0.04)	1.57 (0.18)	0.73 (0.14)	0.66 (0.07)
1/16 oz to 1/8 oz	1.90 (< 0.01)	3.18 (< 0.01)	0.55 (0.02)	0.70 (0.18)
Age (Reference: 18 to 24 years old)				
25 to 34 years old	1.18 (0.65)	1.51 (0.40)	0.34 (< 0.01)	0.62 (0.20)
35 to 54 years old	0.89 (0.81)	1.69 (0.27)	0.39 (< 0.01)	0.55 (0.08)
55 years and older	1.52 (0.24)	1.02 (0.97)	0.40 (0.01)	0.51 (0.07)
Race/ethnicity (Reference: Non-Hispanic White)				
Non-Hispanic Black	0.89 (0.81)	1.73 (0.35)	4.57 (< 0.01)	3.91 (< 0.01)
Hispanic	1.01 (0.98)	1.48 (0.27)	1.13 (0.69)	1.86 (0.04)
Non-Hispanic Other race/ethnicity	1.11 (0.76)	1.27 (0.62)	1.28 (0.49)	0.95 (0.90)
Education (Reference: Less than high school graduation)				
High school graduate	0.96 (0.95)	0.13 (0.04)	1.18 (0.82)	1.50 (0.64)
Some college	0.98 (0.98)	0.23 (0.11)	1.35 (0.67)	1.68 (0.54)
College degree or higher	1.15 (0.84)	0.21 (0.09)	1.86 (0.38)	1.90 (0.45)
Employment (Reference: Not employed)				
Self-employed or employed for wages	2.21 (<0.01)	3.37 (< 0.01)	1.35 (0.14)	1.27 (0.27)
State of Residence (Reference: Washington)				
Lives in Colorado	0.96 (0.80)	1.47 (0.13)	0.89 (0.54)	0.74 (0.12)

^**1**^ Dichotomous indicator for “agree” or “strongly agree.”

Multivariate analysis further showed that increased perceptions that driving high is unsafe was associated with significantly lower odds of agreeing or strongly agreeing with the statement “I might drive even though I know I shouldn’t” (OR = 0.25, *P* < 0.01). Similarly, increased perceptions that driving high is unsafe was associated with significantly lower odds of agreeing or strongly agreeing with the statement “In certain situations, I might drive high” (OR = 0.21, *P* < 0.01). Increased knowledge of the potential legal consequences of driving while high was not significantly associated with either of the openness outcomes (OR = 1.02, *P* = 0.88; OR = 1.23, *P* = 0.09, respectively).

### Association between individual characteristics of marijuana users and marijuana DUI behavior and openness

Increased marijuana consumption was associated with higher odds of any driving while under the influence of marijuana in the past year. Relative to respondents who reported “low use” (1/128 oz. or less) on the last day marijuana was used, those who consumed “moderate” amounts (1/32 oz or 1/64 oz) or “high” amounts (1/8 oz. or 1/16 oz.) on the last day used were more likely to report any driving while under the influence of marijuana in the past year (OR = 1.51, *P* = 0.04, OR = 1.90, *P* < 0.01, respectively). Increased marijuana consumption was also associated with significantly higher odds of reporting in the past month driving 5 or more times within 1 hour of consuming marijuana or hashish (“moderate” consumption OR = 1.57, *P* = 0.18, “high” consumption OR = 3.18, *P* < 0.01). Post-estimation Wald tests confirm that the magnitude of these positive associations with any driving after marijuana use in the past year is significantly larger for the “high” consumption category compared to the “moderate” consumption category, suggesting dose response. We also found that being self-employed or employed for wages was associated with a significantly higher odds of any driving after marijuana use in the past year (OR = 2.21, *P* < 0.01) and a higher odds of reporting in the past month driving 5 or more times within 1 hour of consuming marijuana or hashish (OR = 3.37, *P* < 0.01).

A number of individual characteristics were also associated with openness to driving while high. Relative to those who reported “low use” on the last day marijuana was used, respondents who reported “high use” were significantly less likely to agree or strongly agree with the statement “In certain situations, I might drive high” (OR = 0.55, *P* < 0.02).” Increased marijuana use was not significantly associated with agreement with the statement “I might drive high even though I know I shouldn’t.” We also found that increased age was associated with lower openness to driving while high. Respondents aged 25 to 34 (OR = 0.34, *P* < 0.01), those aged 35 to 54 (OR = 0.39, *P* < 0.01), and those aged 55 and over (OR = 0.40, *P* = 0.01) were significantly less likely than 18 to 24 year old marijuana users to agree or strongly agree with the statement “In certain situations, I might drive high.” The age groups of 35 to 54 (OR = 0.55, *P* = 0.08) and 55 and over (OR = 0.51, *P* = 0.07) were associated with marginally significant decreases in the odds of agreeing or strongly agreeing with the statement “I might drive high even though I know I shouldn’t.”

Openness to driving while high was also associated with race/ethnicity. Non-Hispanic black respondents were significantly more likely than non-Hispanic white respondents to agree or strongly agree with the statement “In certain situations, I might drive high” (OR = 4.57, *P* < 0.01). Non-Hispanic black (OR = 3.91, *P* < 0.01) and Hispanic respondents (OR = 1.86, *P* = 0.04) were significantly more likely than non-Hispanic white respondents to agree or strongly agree with the statement “I might drive high even though I know I shouldn’t.” Lastly, there were no significant differences in any of the behavioral or openness outcomes between respondents in Colorado and Washington.

## Discussion

This study suggests that increased perceptions that driving while under the influence of marijuana is unsafe and knowledge of the legal consequences of driving while high are significantly associated with decreased odds of marijuana DUI behavior. However, the magnitude of this association was significantly larger for safety perceptions compared to knowledge of marijuana DUI laws. We also find that perceptions that driving after marijuana use is unsafe is associated with decreased openness to driving while high in the future. However, knowledge of marijuana DUI laws is not associated with openness to driving while high.

These results have potentially important implications for the implementation of interventions aimed at mitigating the potential unintended consequence of increased marijuana DUI behavior that may arise with marijuana commercialization. As noted previously, marijuana-related driving impairment has been a focal point of early interventions designed to mitigate this consequence, including the recent “Drive High, Get a DUI” campaign that aired in Colorado and Washington. This campaign focused exclusively on knowledge of the legal consequences of driving while high. While our results show that knowledge of marijuana DUI laws is associated with a decreased likelihood of marijuana DUI behavior, it is a weaker predictor of DUI behavior compared with perceptions that driving high is unsafe. In addition, safety perceptions strongly predict decreased openness to driving high while knowledge of DUI laws was not associated with openness. These findings suggest that interventions aimed at reducing the incidence of marijuana DUI are likely to be more successful by targeting safety perceptions related to marijuana DUI rather than knowledge of DUI laws.

One potential explanation for why knowledge of DUI laws was a weaker predictor of marijuana DUI behaviors and openness is that overall knowledge of laws against driving while under the influence of marijuana is very high. The vast majority of our respondents reported a high degree of certainty that they could get a DUI for driving high, lose their license for driving high, or pay a fine for driving high (mean knowledge scale = 4.13 on a scale of 1 to 5). Without pre-legalization data, it is unclear the degree to which the near ceiling levels of knowledge related to marijuana DUI laws is a result of increased public awareness of the implementation of legalized marijuana or the result of public education initiatives like the “Drive High, Get a DUI” campaign. However, the observed ceiling levels of this outcome suggest that it could be not only a weaker predictor of relevant outcomes but also less sensitive to interventions that target it.

Our study also demonstrates a number of other individual-level characteristics of marijuana users that are associated with marijuana DUI behavior and openness to driving high. Unsurprisingly, heavier marijuana consumption was associated with a higher likelihood of driving while high. We also found that while age was not associated with DUI behavior, older age was significantly associated with less openness to driving while high. In addition, we found that being employed was associated with increased driving while under the influence of marijuana. These results may be helpful in developing targeting strategies for future interventions aimed at reducing marijuana DUI. Our findings suggest that marijuana users who are relatively young or who consume greater amounts of marijuana may be at higher risk of marijuana DUI and thus may benefit most from targeted interventions to address this behavior. To the extent that employment is also a marker for increased marijuana DUI, likely via correlations with disposable income, higher-income marijuana users may also be a potential target for future interventions and messages.

The descriptive data presented in this study also highlights several broader concerns for public health and safety. For example, the overall prevalence of driving while under the influence of marijuana in the past year is substantial. More than 40% of respondents reported driving a motor vehicle while feeling the effects of marijuana or hashish at some point in the past year. Additionally, we found that nearly one quarter of all respondents reported driving a motor vehicle more than 5 times in the past 30 days while using marijuana. A recent report published by the Colorado Department of Public Health [14] showed that during late 2013 and early 2014, approximately 18.0% of past 30-day marijuana users in Colorado reported driving a car more than 5 times while using marijuana in the past 30 days. While our measure includes the more general term “motor vehicle,” these results suggest that driving while under the influence of marijuana may be trending upward.

Our data further suggest that many marijuana users in Colorado and Washington believe that driving while under the influence of marijuana or hashish is safe in general and safer than driving under the influence of alcohol. Roughly one-third of respondents in our sample believed they can safely drive while under the influence of marijuana. Furthermore, more than half of respondents believed that it is safer to drive under the influence of marijuana than alcohol. These findings are consistent with previous studies [16, 17, 18] that have indicated marijuana users believe marijuana use impairs driving only slightly whereas attitudes toward drunk driving are more negative. Studies that have attempted to compare the degree of driving impairment between marijuana and alcohol have been inconclusive due to the less predictable effects of marijuana compared with alcohol [19]. However, it is clear that marijuana use impairs the ability to drive [3, 4, 18], particularly among occasional marijuana users who may be less tolerant to THC [5]. Thus while the accuracy of our participants’ perceptions about the comparative safety between marijuana- and alcohol-related driving impairment is unclear, our data suggest there are misperceptions about the general safety of driving while under the influence of marijuana among users in Colorado and Washington.

Another potential concern elucidated by our data is the rate at which marijuana users report openness to driving after using marijuana. About28% of respondents in our data indicate that they might drive high even though they know they should not, and approximately 41% of respondents say they might drive high in certain situations. These results suggest there is measurable willingness to drive while using marijuana in the two states we studied. Based on related behavior change theories [12, 13] that predict that behavioral intent and similar cognitive constructs are antecedent to actual behavior, these patterns of safety misperceptions and willingness to drive while high suggest there is a need for public health messaging to increase awareness about the risks of driving while under the influence of marijuana.

The primary limitation of this study is that the findings are based on an online panel recruited with a convenience sample that is not probability-based. Similar to most online panels, a higher proportion of respondents in our sample were non-Hispanic white and more educated than the general population. As a result, our findings are applicable to the population included in our sample but likely not generalizable to the broader populations of marijuana users in Colorado and Washington. In light of recent trends in marijuana liberalization, future research should consider larger investments in state-level representative survey systems that include rich, detailed information on marijuana behaviors, risk perceptions, and other measures that are not collected in existing national health and drug use surveys. A second limitation is that our results reflect cross-sectional associations between the outcomes measured and independent variables of interest. Therefore, causal relationships between the key independent variables and outcomes measured cannot be assumed. A final limitation is that the perceptions and knowledge measures presented in this study were new constructs that were created to capture what the authors believe are important aspects of marijuana risk perceptions in the context of the new retail markets in each state. While factor analyses demonstrate that our measurement scales for these outcomes had acceptable internal reliability, more research is needed to further test these measures and determine the extent to which they are predictive of marijuana behaviors while driving.

As commercial marijuana markets in Colorado and Washington evolve and as new markets emerge in other states, it is important for these states to develop data-driven strategies for intervening on potential unintended consequences of marijuana liberalization. This entails identifying behavioral precursors and message concepts that are likely to generate change in marijuana DUI behavior (or other behavioral outcomes of interest). Our findings suggest that future interventions aimed at mitigating the potential unintended consequence of increased marijuana DUI should not focus on merely raising awareness and knowledge of legal consequences but rather engage marijuana users to increase perceptions that driving while high is unsafe. In addition, users who consume marijuana more heavily, those who are younger, and those who are non-Hispanic black or Hispanic may benefit most from targeted messages to reduce marijuana DUI. While these results provide practical testing of a knowledge-based approach to reducing marijuana DUI, formal evaluation or efficacy assessments of real-world interventions that use this approach are warranted. These data may provide actionable information and insights on the development of future messages and interventions to protect public safety and mitigate increases in marijuana DUI.

## Ethics Statement

This study included human subjects who participated in a self-administered online survey. All survey instruments and protocols were reviewed and approved by the sanctioned Institutional Review Board of RTI International.

## References

[1] Ferner M. Obama predicts bright future for legal weed. 2015 January 22 [cited 14 July 2015]. Available: http://www.huffingtonpost.com/2015/01/22/obama-marijuana-youtube_n_6527958.html.

[2] Berning A, Compton R, Wochinger K. Results of the 2013–2014 National Roadside Survey of Alcohol and Drug Use by Drivers. Report DOT HS 812 118. Washington, DC: National Highway Traffic Safety Administration. Office of Behavioral Safety Research; 2015.

[3] Robbe HWJ, O’Hanlon JF. Marijuana and actual driving performance. Report No. DOT HS 808 078 Washington, DC: U.S. Department of Transportation; 1993.

[4] Moskowitz H. Marijuana and driving. Accid Anal Prev. 1985 8;17(4): 323–345. 3006718 10.1016/0001-4575(85)90034-x

[5] Hartman RL, Huestis MA. Cannabis effects on driving skills. Clin Chem. 2013 3;59(3): 478–492. 10.1373/clinchem.2012.194381 23220273 PMC3836260

[6] Compton RP, Berning A. Drug and alcohol crash risk (Traffic Safety Facts Research Note, Report Number: DOT HS 812 117) Washington, DC: National Highway Traffic Safety Administration; 2015.

[7] Salomonsen-Sautel S, Min SJ, Sakai JT, Thurstone C, Hopfer C. Trends in fatal motor vehicle crashes before and after marijuana commercialization in Colorado. Drug Alcohol Depend. 2014 7 1;140: 137–144. 10.1016/j.drugalcdep.2014.04.008 24831752 PMC4068732

[8] Couper FJ, Peterson BL. The prevalence of marijuana in suspected impaired driving cases in Washington state. J Anal Toxicol. 2014 10;38(8): 569–574. 10.1093/jat/bku090 25217548

[9] Hughes T. Marijuana 'edibles' pack a wallop. 2014 May 8 [cited 14 July 2015]. Available: http://www.usatoday.com/story/news/nation/2014/05/08/marijuana-pot-edibles-thc-legalized-recreational/8463787/.

[10] Gurman S. Colorado deaths stoke worries about pot edibles. 2014 April 18 [cited 14 July 2015]. Available: http://www.huffingtonpost.com/2014/04/18/pot-deathscolorado_n_5175876.html?ncid=fcbklnkushpmg00000013&ir=Politics.

[11] Monte AA, Zane RD, Heard KJ. The implications of marijuana legalization in Colorado. JAMA. 2015 1 20;313(3): 241–242. 10.1001/jama.2014.17057 25486283 PMC4404298

[12] Bandura A. Social foundations of thought and action: A social cognitive theory Englewood Cliffs, NJ: Prentice-Hall; 1986.

[13] Fishbein MA. Belief, attitude, intention, and behavior Reading, MA: Addison-Wesley; 1975.

[14] Colorado Department of Public Health and Environment. Influential factors in health living: A survey study of selected health conditions and behaviors among Colorado adults 2014 12 [cited 4 June 2015]. Colorado: Community Epidemiology and Program Evaluation Group Available: http://www.ucdenver.edu/academics/colleges/PublicHealth/community/CEPEG/WkProducts/Reports/Documents/120814%20IFHL%20Report%20Final.pdf.

[15] Carmines E.G., Zeller R.A. Reliability and Validity Assessment. Newbury Park, CA: Sage Publications 1979.

[16] Terry P, Wright KA. Self-reported driving behavior and attitudes towards driving under the influence of cannabis among three different user groups in England. Addict Behav. 3 2005;30(3): 619–626. 15718082 10.1016/j.addbeh.2004.08.007

[17] Kelly E, Darke S, Ross J. A reivew of drug use and driving: epidemiology, impairment, risk factors and risk perceptions. Drug Alcohol Rev. 2004;23(3): 319–344. 15370012 10.1080/09595230412331289482

[18] Danton K, Misselke L, Bacon R, Done J. Attitudes of young people toward driving after smoking cannabis or after drinking alcohol. Health Educ J. 2003;62: 50–60.

[19] Sewell RA, Poling J, Sofuoglu M. The effect of cannabis compared with alcohol on driving. Am J Addict. 2009;18(3): 185–193. 10.1080/10550490902786934 19340636 PMC2722956

